# The Pharmacopœia of the United States of America

**Published:** 1842-04-30

**Authors:** 


### BIBLIOGRAPHICAL NOTICE.

THE PHARMACOPŒIA OF THE UNITED STATES OF AMERICA. *By Authority of the National Medical Convention, held at Washington, A. D.* 1840. Philadelphia. Grigg & Elliot, 1842. 8vo. pp. 279.

The present is the third edition of the Pharmacopœia of the United States. It is published under the auspices of a committee of revision and publication, appointed by the National Medical Convention held at Washington, in January, 1840*, consisting of Professors Wood, Bache, and Dunglison, of Philadelphia, Drs. Cohen, of Baltimore, Dunn, of Newport, Stevens, of Savan-

* The official account of the proceedings of the Convention will be found at page 58, vol. 3d, of the Examiner.

nah, and Sewall, of Washington. The practical duties of the committee, which met in Philadelphia, devolved upon the three first named gentlemen, owing to the necessary absence of the remaining members. The publication of the Pharmacopœia has been delayed for more than a year to enable the committee to avail itself of the co-operation of the Philadelphia College of Pharmacy. A committee of that body submitted a revised copy of the Pharmacopœia, "elaborated with ability and great industry, and presenting, along with numerous individual additions and alterations, some new features in the general plan." This induced the committee of publication, after having already carefully revised the entire work, again to go over the whole ground. As the alterations determined upon were too numerous to admit of incorporation with the existing Pharmacopœia, it became necessary that it should be wholly re-written. These causes, which greatly enhance the value of the work, have postponed its publication to the present time.

We proceed to notice the principal modifications which have been introduced into the present edition of the Pharmacopœia. The excellent system of nomenclature, adopted in the two first editions, has been retained in the present. A few changes have been made in the names to avoid confusion in products of similar origin, as *Amygdala Amara* and *Amygdala Dulcis* for *Amygdala*; or, to bring the names in accordance with the present state of science, as *Cetraria* for *Lichen*, *Tinct. Ferri Chloridi* for *Tinct. Ferri Muriatis*, &c.

"The point in which the present edition of the Pharomacopœia most strikingly differs from the preceding is, in being exclusively in English. There seems to be no sufficient practical advantage to counterbalance the inconvenience of attempting to present ideas in a language which has no appropriate words to express them, and the labour and expense incurred in printing twice as much matter as is necessary to convey the meaning intended. The recent publication, moreover, of the French Codex and the Edinburgh Pharmacopœia, in the vernacular languages of the two countries respectively, gives the sanction of high authority to the course now pursued. But, though English is used exclusively in all the directions and explanations of the U. S. Pharmacopœia, it has been deemed best to retain the Latin names of medicines, as these are still very generally and advantageously employed in prescriptions. Another novel feature of the present edition is the introduction, in connection with certain articles of the Materia Medica and certain preparations, of brief notes, indicating the readiest means of ascertaining their genuineness and purity. It is only to the substances of mineral origin, and those which are the result of chemical processes, that these notes have been appended. In relation to the characteristic properties of organic substancces, the means of judging are less precise, and cannot be so readily expressed in a few brief rules."—*Preface*, pp. 16, 17.

In the arrangement of the *Materia Medica*, in the present edition, the Latin and English titles are followed by a brief statement, answering the purpose of a definition, and, in general, by a note indicating the means of testing the genuineness and purity of the medicine. A secondary list of medicines, little employed or of doubtful value, is retained as in former editions. On the present occasion, several medicines, as *Calamus*, *Cimicifuga*, and *Lactucarium*, have been transferred from the secondary to the primary list, and others, as *Iris Florentina* and *Mucuna*, from the latter to the former. A number have been introduced into both lists, and a few discarded as useless. Among the former are *Creasote*, *Diosma* or *Buchu*, *Oil of Cubebs*, and others of less importance. A large number of new *preparations* are introduced, which, for the most part, had been sanctioned by general use. This department of the Pharmacopœia, as the most important, has, the committee state, received a proportionate share of attention. In the selections of formulæ for new preparations, the processes of the London or Edinburgh College, when wholly approved, have been adopted from a desire to promote uniformity in countries using the same language. For many of the preparations two distinct processes are given,—that is, in cases to which the mode of filtration denominated *displacement* is adapted, this duplication is introduced. Displacement affords great advantages in an economical point of view, and in the character of the resulting preparations, but it requires skill and experience in its proper arrangement. We extract at length the formula recommended by the Pharmacopœia for this important process.

"The kind of filtration commonly called *displacement*, which is employed in many of the processes of this Pharmacopœia, is to be effected in the following manner, unless otherwise specially directed. A hollow cylindrical instrument is to be used, somewhat conical towards the inferior extremity, having a funnel-shaped termination so as to admit of being inserted into the mouth of a bottle, and provided internally, near the lower end, with a transverse partition or diaphragm pierced with numerous minute holes, or, in the absence of such a partition, obstructed with some insoluble and inert substance, in such a manner that a liquid poured into the cylinder may percolate slowly.

The substance to be acted upon, having been reduced to a coarse powder, and mixed with enough of the menstruum to moisten it thoroughly, is, after a maceration of some hours, to be introduced into the instrument, and slightly compressed upon the diaphragm. Any portion of the macerating liquid which may not have been absorbed by the powder, is afterwards to be poured upon the mass in the instrument, and allowed to percolate. Sufficient of the menstruum is then to be gradually added to drive before it, or displace the liquid contained in the mass; the portion introduced is in like manner to be displaced by another portion; and so on till the required quantity of filtered liquor is obtained. If the liquor which first passes should be turbid, it is to be again introduced into the instrument. Care must be taken that the powder be not, on the one hand, too coarse or loosely pressed, lest it should allow the liquid to pass too quickly, nor, on the other, too fine or compact, lest it should offer an unnecessary resistance. Should the liquor flow too rapidly, it is to be returned to the instrument, which is then to be closed beneath for a time, in order that the finer parts of the powder may subside, and thus cause a slower percolation.

